# Iron Status and Physical Performance in Athletes

**DOI:** 10.3390/life13102007

**Published:** 2023-10-02

**Authors:** Andrea Solberg, Håkon Reikvam

**Affiliations:** 1 Faculty of Medicine, University of Bergen, 5007 Bergen, Norway; andrea.solberg@student.uib.no; 2 Institute of Clinical Science, Faculty of Medicine, University of Bergen, 5007 Bergen, Norway; 3 Clinic for Medicine, Haukeland University Hospital, 5009 Bergen, Norway

**Keywords:** iron, athletes, sport performance, iron supplementation

## Abstract

Iron is an important mineral in the body, essential for muscle function and oxygen transport. Adequate levels of iron in the blood are necessary for athletes, as iron-deficiency anemia can reduce physical performance. Several studies have investigated iron status and supplementation in iron-deficient athletes, and determined how physical strain can change iron balance and markers related to iron status. The question of how to influence and optimize iron status, as well as other markers that can affect iron metabolism, has been less thoroughly investigated. Therefore, the aim of this review is to take a closer look at the importance of iron values, iron markers, and factors that can change iron metabolism for physical performance and the extent to which physical performance can be influenced in a positive or negative way. A systematic search of the PubMed database was performed, with the use of « iron» or «iron deficiency» or «hemoglobin» AND «athletes» AND «athletic performance» as a strategy of the search. After the search, 11 articles were included in the review after the application of inclusion and exclusion criteria. Major findings include that iron supplementation had the best effect in athletes with the lowest iron status, and effects on physical performance were mostly achieved in those who were originally in a deficit. Iron supplementation could be beneficial for optimal erythropoietic response during altitude training, even in athletes with normal iron stores at baseline, but should be performed with caution. Alteration of the hepcidin response can affect the use of existing iron stores for erythropoiesis. Energy intake, and the amount of carbohydrates available, may have an impact on the post-exercise hepcidin response. Optimal vitamin D and B12 levels can possibly contribute to improved iron status and, hence, the avoidance of anemia.

## 1. Introduction

Iron is an essential mineral; its main tasks are reversibly carrying oxygen in hemoglobin molecules in red blood cells and in myoglobin in muscle cells. It has other important roles, such as contributing to the electron transport chain, enzymes, DNA synthesis, and energy metabolism [1,2]. Iron is taken up by cells and transported to mitochondria where it can be used to form heme (iron bound to porphyrin), which is the form of iron included in hemoglobin and myoglobin molecules [3]. Iron’s chemistry is important in biological processes because its ions can give away or accept electrons. It can facilitate redox reactions by working as a cofactor for different enzymes and proteins [2]. Iron can also be toxic at high levels because its redox capacity can contribute to the formation of reactive oxygen species (ROS), which can cause cell damage and cell death [4]. To avoid unwanted reactions, iron is transferred in the blood with the transport protein, transferrin [5], and excess iron is stored as ferritin in the liver and the reticuloendothelial system [1,5]. Ferritin is the most used biomarker for iron status [6]. Most of the iron in the body is actively used in hemoglobin, myoglobin, and enzymes. Iron storage is approximately 4 g of iron in men and 2.5 g of iron in women, but only 1–2 mg is lost per day due to intestinal iron absorption and the efficient recycling system of iron [1,7]. Normal intake of iron is around 10–15 mg each day, but only 10% is absorbed under normal conditions, when iron losses only occur in small doses due to epithelial desquamation and minor bleeding [7].

Iron balance in the blood is carefully regulated by the peptide hormone, hepcidin, which is produced by the hepatocytes in the liver [8]. Hepcidin is upregulated when serum ferritin is high and is regulated by the need for iron for erythropoiesis. Hepcidin inhibits the absorption of iron by binding to its receptor on ferroportin that transports Fe^2+^ from enterocytes to plasma [7,8]. Hepcidin production has also been shown to be stimulated by inflammatory markers such as interleukin-6 (IL-6), which is upregulated with the inflammation response happening after training [9,10,11]. This response is also dependent on the baseline levels of ferritin before training; athletes with lower ferritin levels have a lower hepcidin response after training [11,12]. Recent studies have focused on a possible altered hepcidin response with low energy availability (LEA) and a low-carbohydrate or ketogenic diet [13,14,15]. McKay et al. highlighted, in their review, an increased hepcidin response under both LEA and low-carbohydrate conditions in athletes [15].

Iron deficiency can occur with or without anemia [1,16,17,18]. Iron deficiency without anemia (IDNA) is diagnosed when ferritin levels are low (<30 mg/L) but hemoglobin levels are normal (>130/120 g/L in men/women). Iron deficiency with anemia (IDA) occurs when low ferritin levels lead to low levels of hemoglobin (<130/120 g/L in men/women), defined by WHO [16,17]. A ferritin cut line of 30 mg/L is normal for adults, but it can vary, and the optimal level of ferritin for athletes is highly debated [19,20,21,22]. Because of iron’s role in biological processes, outside of carrying oxygen in hemoglobin molecules, IDNA may have a negative impact on multiple functions. Metabolic systems with iron-containing proteins can be affected by IDNA itself, such as reactions in the respiratory chain where iron works as a cofactor, thereby reducing oxidative capacity, which again reduces the muscles’ ability to use oxygen [1,18,23]. Symptoms such as fatigue, reduced concentration, and impaired physical performance can occur with IDNA [17,18]. When IDA occurs, the oxygen-carrying capability in the blood is reduced because of lower hemoglobin levels. This reduces physical capabilities because of lack of oxygen to all cells in the body, including those of working muscles during exercise [24]. A reduction in VO_2max_ and endurance capacity is likely to appear [25,26], whilst supplementation of iron in IDA athletes can contribute to an increase in hemoglobin and, thereby also an increase in VO_2max_ and endurance capacity [26,27].

Iron deficiency in athletes is normal, and they are prone to it due to several mechanisms, including increased losses of iron during training caused by micro-ischemia, hemolysis, sweating, etc. Furthermore, women are more prone than men because of menstrual bleeding [1,11,28]. A link between low energy availability (LEA) and poor iron status in athletes is likely, as the dietary intake of iron may not be sufficient [29]. The inflammatory response that occurs due to training, with increased IL-6 and hepcidin levels, opens a window where less iron is absorbed and recycled [9,24]. In addition, there is an increased utilization of iron for the increased erythropoiesis and rebuilding processes that occur as a result of training. Training and living at altitude, or in hypoxic environments, leads to an increase in hemoglobin following an increase in erythropoietin (EPO) production [30,31]. A rise in EPO increases erythropoiesis: the production of red blood cells in the bone marrow. Living and/or training at altitude is a widely used regime for endurance athletes to increase the oxygen-carrying capacity of their blood and their endurance capabilities [11,30,31]. Hematological adaptations to hypoxia are dependent on adequate iron stores. Ferritin values > 50 ng/mL are recommended prior to altitude training, due to the increased need of iron in these environments [1]. Okazaki et al. showed, both in a retrospective study and a prospective study, that iron deficiency inhibited erythropoietic responses to altitude training and highlighted the importance of iron supplementation when aiming for an optimal adaptation to altitude training [32]. Iron deficiency can affect several of the abilities that athletes need to perform outside of aerobic abilities, including those related to strength, the immune system, fatigue, and mood status [24]. All of these factors can affect endurance, as well as power, speed, coordination, concentration, recovery, and consequently, performance in various sports variables. Notably, endurance athletes are a frequently studied group when it comes to iron deficiency, because of iron’s role in aerobic metabolism, and the high prevalence of iron deficiency in endurance athletes [33].

Given iron’s crucial biological role and its impact on performance when deficiency occurs, there are still conflicting findings and debates about how to recommend optimizing iron status for athletes. There are no clear recommendations on how to supplement iron in different forms, monitor blood markers, and adjust other factors that affect iron status. Optimizing these factors may have an impact on how athletes, both with and without iron deficiency, can improve their physical performance. The aims of this study were to investigate the importance of iron status, and factors that can alter iron metabolism for physical performance. It also aimed to determine to what extent physical performance can be influenced positively or negatively in relation to iron status. Furthermore, it sought to determine whether only athletes with iron deficiency experience improved effects with better iron status or if there is room for optimization regardless of the athletes’ initial iron status.

- Which blood markers should be emphasized to optimize an athlete’s iron status and prevent its reduction?
- What is the significance of iron supplementation for physical performance in athletes with iron deficiency compared to those with sufficient iron stores? With iron deficiency vs. sufficient iron stores?
- Is it sufficient to aim for normal iron status in athletes, or can we optimize further by using more specific reference ranges or other markers?

## 2. Materials and Methods

This study followed the guidelines of Preferred Reporting Items for Systematic Reviews and Meta-Analyses (PRISMA) [34].

A structured literature search was performed using the PubMed database. The following terms were included in the search: “Hemoglobins” or “Iron” or “Iron Deficiencies” or “Hepcidins” or “Ferritins” or “Transferrins” or “Hemoglobin”, AND Athletes or “Athletic performance”. The algorithm for the complete advanced search performed in PubMed is presented in the Supplementary Materials. The search was limited to the last 10 years (2013–2023), and to younger athletes aged 19–44 years old. Out of the advanced search, 410 articles were reviewed for selection, based on title and abstract. The following inclusion criteria were applied:

- Date of publication between 2013–2023;
- Written in English;
- Healthy athletes between 19–44 years of age;
- Only original research articles, no reviews or meta-analysis;
- Including studies of iron’s influence on physical performance, which is required beyond ordinary physical activity in the general population. This includes athletes training with the intention of improving physical performance, and those with higher workload demands for performance enhancement, beyond what is necessary for survival.

This excludes studies related to the influence on iron status due to illness, old age, etc. Studies dealing with changes in iron metabolism because of exercise or factors surrounding exercise, but not directly related to iron status and its correlation to physical performance, were also excluded. 

**Figure 1:**
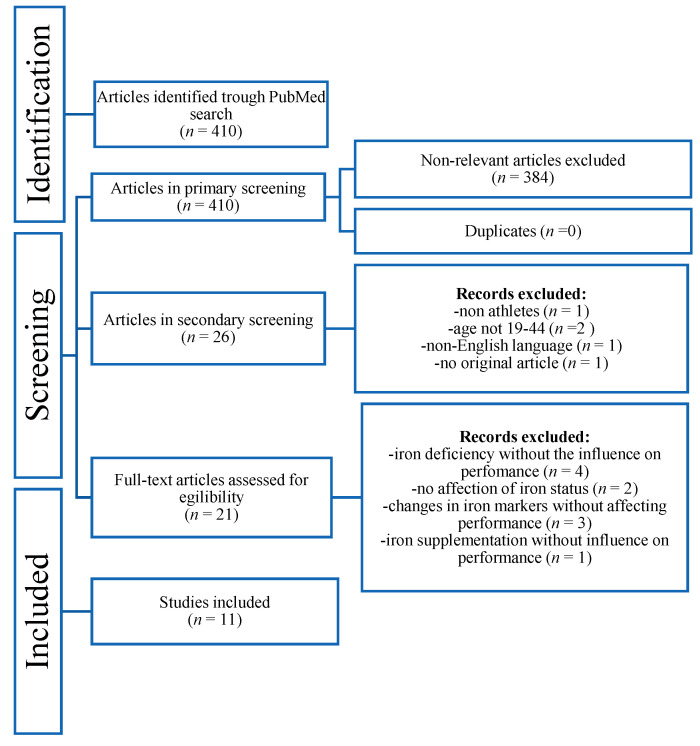
Flow chart of the inclusion/exclusion process of the systematic review.

Out of the 410 articles obtained from the initial search, 384 were excluded, not meeting the present inclusion criteria. Duplicates were checked through EndNote (0), and 26 articles were retained for full review. Of these, 2 were excluded for age, 1 for being non-English-language, 1 for being centered around non-athletes, and 1 for not being an original article. Additionally, 10 articles were excluded for not involving iron’s correlation to physical performance. Hence, 11 articles met the inclusion criteria for this systematic review. The flow diagram of the inclusion/exclusion process is illustrated in Figure 1. A brief overview of the articles included is given in Table 1.

## 3. Results

### 3.1. Literature Review 

From the articles included, the following information was obtained: authors, sample size and type of sport, population characteristics, experimental protocol (including aim, interventions, cut off values), main outcome, and potential effects on physical performance. The key findings from the 11 articles are summarized in Table 1.

### 3.2. Population Characteristics

Among the 11 studies in the present review, a total of 109 women, 139 men, and 284 of unknown gender were included. All of them were athletes, with the majority being endurance athletes: 118 were identified as runners, 18 as cyclists and 76 as rowers. Other sports included athletes competing in volleyball, triathlon, and track and field with unknown specificity (comprising both strength and endurance athletes). 

### 3.3. Studied Factors and Parameters

Six of the articles examined the effects of oral iron supplementation [32,35,38,39,40,41], while two also included intravenous supplementation, either alone [40], or in combination with oral supplementation for comparison [38]. Doses between 44 and 325 mg/day were given orally, either fixed doses, or based on serum ferritin values. 100–200 mg/day were given in those with intravenous supplementation. To evaluate the impact of supplementation, various blood parameters were primarily considered, including serum ferritin, serum iron, hemoglobin, hematocrit, transferrin, RCV, MCV, MCH, and hepcidin. In terms of performance, assessment were made using measurements such as VO_2max_, VO_2peak_, energetic efficiency, lactate response, time trials, mood and fatigue, as well as strength tests. 

In three of the included articles, supplementation was administered to athletes classified as iron-deficient [32,39,41], using a serum ferritin cutoff between 20–30 ng/mL (20 ng/mL for women, 30 ng/mL for men). They also provided supplementation to iron-sufficient athletes simultaneously, classifying the athletes into their respective category during the study. Six studies gave supplementation to athletes considered iron-sufficient, with three of them only to athletes who were not in a deficiency [35,38,40]. Using the cut of serum ferritin > 30 ng/mL, but also setting the values between 30–99 ng/mL as functional iron deficiency, with only serum ferritin > 100 ng/mL considered as sufficient [41]. Two studied the effect of supplementation in altitude [32,38], or in altitude simulation (hypoxic environment). Meanwhile, one study investigated the impact on post-exercise inflammation (IL-6 and hepcidin) and iron response, with recovery in a hypoxic environment [42].

One article looked at the differences in iron absorption and hepcidin response when training and consuming standardized meals at different times during the day [37]. Considering the diurnal variation in hepcidin and the acute post-exercise inflammation phase, which can influence iron absorption. Two articles looked at vitamin D and its correlation with iron status with intervention of supplementation of 3000 or 10,000 IU/day. Kasprowicz et al. investigated baseline vitamin D status and compared post-exercise inflammation responses on iron [36]. While Mielgo-Ayuso et al. assessed vitamin D supplementation on iron and hemoglobin response, as well as cortisol testosterone response, for muscle recovery status [44]. Both studies used a cut-off 25(OH)D < 30 ng/mL for vitamin D deficiency. Krzywánsk et al. collected 1131 blood samples over six years from 243 track and field athletes, investigating vitamin B12 status and its correlation to red blood cell values [43], using a cut-off of <197 pg/mL for vitamin B12 deficiency. The average vitamin B12 level was 739 pg/mL in strength athletes, and 881 pg/mL in endurance athletes. 34% of the athletes used vitamin B12 supplementations.

### 3.4. Effect of Iron Supplementation

Out of the six studies looking at iron supplementation, two highlighted the importance of sufficient iron stores prior to and during altitude exposure [32,38]. In the retrospective study by Okazaki et al., only those with normal ferritin levels had improved VO_2max_, while those with low ferritin levels showed no improvement in VO_2max_ after four weeks of altitude training. In the prospective study, VO_2max_ increased in athletes training at altitude and supplementing with iron simultaneously. Whereas those who trained at sea level with iron supplement did not improve [32]. Garvican-Lewis et al. reported improved VO_2peak_ for athletes receiving intravenous iron supplementation during LHTL simulation [38]. A significant increase in hemoglobin was seen in athletes given both IV iron and oral iron supplementation (3.7% and 3.2%), while those given placebo had no increase in hemoglobin. Both studies demonstrated that iron supplementation is necessary for normal ferritin levels when training in a hypoxic environment, even in those with sufficient levels prior to altitude, pointing out the increased iron utilization for the accelerated erythropoiesis under these circumstances. Okazaki et al. calculated an extra need for 4.9 mg iron a day for runners in altitude, in addition to the daily requirements for runners: 1.9 mg/day for women and 2.3 mg/day for men when training at sea level [32].

Only two of the other studies, in which supplementation was given to athletes in non-altitude environments, showed a small increase in physical performance: DellaValle et al. showed improved energetic efficiency and lactate response [39], with the effect being more pronounced in those who had lower ferritin levels. Mielgo-Ayuso et al. found increased strength numbers during the period of iron supplementation [41]. None of the other studies demonstrated a direct increase in physical performance when providing iron to iron-sufficient athletes supplementation in normoxic environments. In these studies, supplementation could prevent a decline in iron stores due to hard physical strain and have positive effects on mood and fatigue [35,40], while a focus on increased risk of decline in iron stores during competitive seasons and endurance training. Mielgo-Ayuso et al. showed that the benefits of an 11-week iron supplementation would not last during the remaining 8 weeks of the season in female volleyball players. They suggested sustaining iron supplementation throughout the season to prevent a decline in iron stores but recommended using regular blood assessments to determine the need for iron supplementation and to avoid toxicity [41].

### 3.5. Effect of Timing Exercise and Iron Intake on Iron Status

McCormick et al. reported better iron absorption after exercise in the morning compared to the afternoon and at a rested state [37]. They emphasized a diurnal tendency of increased and accumulated hepcidin levels in the afternoon, suggesting a possible transient post-exercise mechanism that promotes iron absorption more effectively in the morning. This was noted despite the increase in hepcidin occurring 3 h after training, regardless of whether the training took place in the morning or afternoon. 

### 3.6. Effect of Hypoxic Recovery on Iron Status

Badenhorst et al. found lower levels of hepcidin response in runners who recovered 3 h in a hypoxic environment, compared to those who recovered in a normoxic environment [42]. This suggests that hypoxic recovery could serve as an intervention to increase acute iron absorption from the diet. 

### 3.7. Effect of Vitamin D Supplementation on Iron Status

Kasprowicz et al. showed that high doses of vitamin D could prevent a decline in iron levels as a consequence of training [36]. Meanwhile, Mielgo-Ayuso et al. showed that vitamin D supplementation could prevent a decline in hemoglobin, hematocrit, and a transferrin, potentially contributing to better oxygen transport [44]. Both studies noted a presumed connection between vitamin D and erythropoiesis, given that there could be a correlation between vitamin D and EPO in stem cells, with a subsequent increase in erythropoiesis [45]. Both studies also observed the coexistence of anemia and vitamin D deficiency [46], assuming that vitamin D supplementation could prevent anemia due to its potential inhibition of hepcidin [47,48]. However, Kasprowizc et al. highlighted conflicting results regarding hepcidin response to vitamin D supplementation, as their trial showed no difference in hepcidin response. They suggested a possible dose-dependent response on hepcidin and that vitamin D can influence iron levels after training through other mechanisms that do not affect the hepcidin response [36].

### 3.8. Vitamin B12 Status Influence on Iron Status

Krzywánsk et al. discovered a significant relationship between vitamin B12 and hemoglobin [43]. Hemoglobin increased from low vitamin B12 values until 400 pg/mL, showing no further increase beyond 700 pg/mL. Athletes using B12 injections had higher levels of hemoglobin and hematocrit. Since hemoglobin is an important marker for red blood cell status, they recommended serum levels of vitamin B12 between 400–700 pg/mL for optimal hemoglobin levels, with regular monitoring to consider supplementation when vitamin B12 levels are <400 pg/mL. 

## 4. Discussion

### 4.1. Treatment of Iron Deficiency

The first step in optimizing iron status in athletes is to correct existing iron deficiencies. Treatment of iron deficiency in sports can be accomplished through dietary adjustments, oral supplementation, or supplementation intravenously/intramuscularly [1]. Because of the macrophages efficient recycling of old erythrocytes, only 5–10% of lost iron needs to be replaced by the diet under normal conditions [11,49]. For athletes, it is important to include meat, fish, whole grains, and green vegetables in their diet. Food rich in vitamin C can increase iron absorption, while polyphenols found in coffee, tea and certain plants, can inhibit iron absorption [49]. When iron deficiency occurs, correcting the diet is normally the first step, aiming for 14 mg of iron ingested each day [1]. 

Recently, there have been studies indicating that an athlete’s daily energy intake can affect the hepcidin response and iron absorption after physical activity [15,50]. Low energy availability (LEA) and poor iron status are known phenomena in athletes [51,52]. However, research is being conducted to investigate the connection between carbohydrate availability and hepcidin responses [15,53,54]. The amount of carbohydrates can be limited in athletes with high training loads, or due to low-carb and high fat, i.e., ketogenic diet trends [15,29,55]. McKay et al. highlighted an increased hepcidin response with both LEA and during low carbohydrate availability, as well as the difference between acute carbohydrate restriction and long-term carbohydrate restriction on hepcidin response and iron status [15]. They concluded that the acute effects could influence hepcidin responses, while the long-term approach depended more on the athlete’s original iron status, rather than the carbohydrate intake over time. A moderate carbohydrate intake should be sufficient to control the factors known to affect iron status, but this exact topic requires more research. 

More studies are examining the athletes’ total energy intake, linking LEA to an increased hepcidin response [13,14,29]. Hennigar et al. found an increased hepcidin response and lower iron absorption after training in athletes experiencing energy deficiency compared to those with a normal energy intake [14]. Additionally, Barney and coworkers observed that a prolonged bout of running increased hepcidin and decreased dietary iron absorption compared to rest in trained runners with low iron stores [56]. This suggests that maintaining a sufficient energy balance is crucial to prevent an elevated hepcidin response from training, and thereby reduce dietary iron absorption. When correcting the diet of iron-deficient athletes, providing adequate energy and carbohydrates to match the training demands may be a key factor in optimizing athletes iron status through their diet. 

Dietary correction, along with oral supplementation, is common in athletes with iron deficiency. However, oral supplementation needs to be undertaken with caution due to its possible side effects, as well as the potential for an increase in hepcidin levels as a response to elevated iron levels in the body [57]. Ishibashi et al. found increased hepcidin levels in athletes taking a moderate dose (24 mg/day) of iron over three consecutive days of running, which amplifies the need for caution in iron supplementation, especially in athletes who are not iron-deficient [58]. For athletes with an iron deficiency, a dose between 40–60 mg of elementary iron daily is recommended [1]. Hall et al. compared a single dose (1 × 200 mg) of iron and a split dose (2 × 100 mg) in elite runners at a three-week altitude training camp [59]. The single dose significantly increased hemoglobin mass more than the split doses, and resulted in a lower hepcidin response compared to the split dose. In this case, a single daily dose of iron was found to be more optimal for increasing hemoglobin during altitude training, compared to a split dose. 

Treatment should be carried out carefully and periodically. It is recommended to repeat blood tests after 6–8 weeks to assess the efficacy of interventions. Subsequently, decisions can be made regarding the continuation of treatment to maintain ferritin levels within the normal range [1] and to avoid possible undesired hepcidin responses resulting from iron supplementation. Mielgo-Ayuso et al. found that the benefits of an 11-week iron supplementation protocol in female volleyball players did not lead to the maintenance of iron levels beyond the remaining 8 weeks of the season, after discontinuing iron supplementation [41]. Periodic treatment with iron, balancing to the risk of iron deficiency, undesired hepcidin responses, and potential toxic side effects, underscores the importance of monitoring iron status in athletes, particularly endurance athletes. Athletes are advised to regularly monitor their iron status and take immediate action if deficiency occurs [1,22,24]. Clarke et al. observed decreasing levels of ferritin during the season in female rugby players, with 23% having ferritin levels lower than 30 mg/L, which mostly occurred in the middle of the season [60]. They suggested hematological reviews every six months for women and annually for men, unless other clinical indications are present. However, there is no fixed ferritin level at which iron supplementation is recommended for athletes above the values set for iron deficiency [61]. It could be useful to determine optimal ferritin values for when supplementation is beneficial for athletes. Additionally, clearer guidelines regarding iron supplementation and concerns around increased hepcidin responses resulting from it are needed in order to enhance physical performance. 

Side effects of oral iron supplementation can include gastrointestinal complaints such as nausea, abdominal pain, and constipation [62]. These side effects must be taken into account, as they can impact daily performance. Ferrous sulphate is the most used oral supplementation, together with ferrous fumarate and gluconate [63]. Intravenous supplementation of iron may be considered if ferritin values do not increase with oral supplementation, in the case of non-tolerance of treatment, or if rapid restoration is required [1]. Intravenous iron is more available now because of the carbohydrate skeleton which iron is stabilized by [64]. This allows for the administration of large iron doses without going through the digestive tract. However, noteworthy the pharmacokinetics of these two methods are quite different and may lead to distinct responses in hepcidin regulation. Girelli et al. suggested that the hepcidin response to these two types of ferroportin channels could be different, possibly resulting in greater inhibition in the intestine than in macrophages [65]. This implies a potentially more favorable impact on iron status with intravenous iron supplementation. Garvican-Lewis et al. demonstrated that iron supplementation plays an important role in optimizing erythropoietic adaptation under “live high- train low” environments [38]. However, intravenous iron supplementation showed no extra benefits compared to oral supplementation in terms of athletes’ iron status. 

### 4.2. Iron Deficiency vs. Iron Sufficiency

As IDA has a significant impact on performance, several studies have tried to investigate the consequences of IDNA. Giving iron supplementation to IDNA athletes to evaluate the effects on physical performance, and to determine the impact a lower iron status in athletes can have, without having anemia [33,39,66,67]. Transport of oxygen should not be affected with IDNA, because there should be sufficient levels of hemoglobin to facilitate oxygen transport. However, negative effects of IDNA may affect the functions of enzymes and proteins in the respiratory system, potential leading to reduction in aerobic performance [67]. Nevertheless, there is evidence of both negative and neutral effects on performance in IDNA individuals [33]. Burden et al. conducted a systematic review with meta-analysis (17 studies, 443 participants), to examine the impact of iron supplementation on hematological status and VO_2max_ in IDNA endurance athletes [66]. Iron supplementation had a moderate effect on hemoglobin and VO_2max_, and a significant effect on ferritin levels. DellaValle et al. found improved ferritin levels, energetic efficiency, and lactate response during endurance exercise, in a RCT in IDNA rowers (*n* = 40 women). They suggested that iron supplementation could enhance the benefits of endurance training [39]. On the other hand, Rubeor et al. concluded, based on a systematic review of 12 studies involving 283 participants, that iron supplementation did not improve performance in 50% of the IDNA athletes, using a ferritin cut of <20 µg/L [33]. They assessed performance using parameters such as run trials and time to fatigue, rather than solely relying on VO_2max_, which makes interpretation of the results different compared to Burden et al. 

Studies have also been conducted in athletes exposed to high physical loads, with initially normal iron stores [35,40,68,69,70,71]. Supplementation was given to prevent a decline in iron stores and to observe possible effects on performance. Endurance athletes may be at risk of having a prelatent iron deficiency, as Nielsen et al. discussed in their review [71]. There are conflicting results regarding whether supplementation could improve performance in iron-sufficient athletes, with a possible increase in hepcidin response as a causal link. Both Karamizrak et al. and Newhouse et al. found no improvement in work capacity in athletes engaged in different sports when given iron supplementation in athletes considered prelatent iron-deficient [68,69], while Magazanik et al. found improved VO_2max_ in athletes receiving iron supplementation and a reduction in ferritin levels in the placebo group [70]. Woods et al. administrated intravenous iron to distance runners with ferritin levels between 30–90 ng/mL but found no improvements in time trials or physical performance but reported improved mood and reduced fatigue [40]. When comparing the importance of iron supplementation in iron-deficient versus iron-sufficient athletes in this review, clear physical improvements were observed in supplemented athletes with baseline ferritin levels below 30 ng/mL. This confirms the importance of monitoring and providing supplementation to athletes who are susceptible to iron deficiency. With regard to finding out the effects iron supplementation can have on athletes with ferritin levels above 30 ng/mL, no major results stand out here, although positive correlations were found. The improved mood and reduced fatigue reported by Woods et al. [40] can have substantial impacts on the athletes’ quality of training. Positive attitudes towards the work that needs to be carried out, and having energy to do it, will automatically increase the quality of athletes’ effort, hence increasing physical gains. The increased energy efficiency and lactate response in Della Valle et al. RCT [39] is the only study in this review that reported improvements in endurance with iron supplementation, even among iron-sufficient athletes under normoxic conditions. A small increase in strength was discovered in a study by Mielgo-Ayuso et al. on female volleyball players when they received iron supplementation, indicating greater physical improvements [41]. Since there are not many studies that have investigated the effect of iron supplementation in iron-sufficient athletes, it would be intriguing to expand the research on this topic, emphasizing the need for additional studies to be performed, including exploring the possible increased hepcidin response and weighing the cost–benefit ratio. The impact of iron supplementation on both iron-deficient and iron-sufficient athletes remains debated and inconclusive, although lower ferritin levels at baseline appear to yield a more favorable response in terms of physical performance.

### 4.3. Optimization of Iron Status 

Most of the studies in this review used a selection of standard biomarkers of iron status. The most frequently used were ferritin, hemoglobin, sTfR, TSAT, and hematocrit, as well as hepcidin and IL-6 in studies that examined changes in these markers [36,37,42]. Establishing an optimal or potentially a narrower range of ferritin levels in different sport variables would be relevant for optimal performance. This is a discussed theme, but no clear guidelines have yet been established [19,20,21,22]. Okazaki et al. recommended serum ferritin values of 40–90 ng/mL before altitude training, using a cut-off for iron deficiency of 20/30 ng/mL for women/men [32]. Della Valle et al. used the same cut-off of 20 ng/mL for female rowers [39], while Woods et al. set normal ferritin values between 30–100 ng/mL [40]. Mielgo-Ayuso et al. set a higher standard with only ferritin levels above 100 ng/mL deemed sufficient, 30–99 ng/mL as functionally deficient (FID), and below 30 ng/mL as absolute iron deficiency [41]. Further studies on how FID affects athletes, including evaluating its possible effects on performance, should be conducted. Gafter-Gvili et al. claim that under FID conditions, erythropoiesis is inhibited even when iron stores are considered normal because the iron is not available for erythropoiesis due to elevated hepcidin levels [72]. This condition can occur with chronic diseases, especially diseases involving inflammation [73,74], where the inflammatory cytokine, IL-6, can lead to increases in hepcidin levels. Whether this occurs with the inflammation resulting from exercise is unknown. It would be interesting to see more studies on the altered hepcidin responses under these conditions, to determine if iron becomes more available for erythropoiesis when only altering hepcidin levels with the same amount of iron in the body. Badenhorst et al. found a suppressed hepcidin response after training, even with a normal IL-6 response, when the recovery phase occurred in a hypoxic environment compared to a normoxic environment [42]. Several studies have investigated the effect of hypoxia on hepcidin response [12,32,38,42,75,76]. Some have suggested hypoxia-inducible factor (HIF) to be an iron regulator during hypoxic stimulus and in conditions involving iron deficiency [77]. HIF-activation has been shown to reduce hepcidin levels in mice [77], but other mechanisms such as EPO/erythropoiesis [78,79] or iron signaling pathways have also been shown to trigger the downregulation of hepcidin [80,81]. Hennigar et al. even found a suppressed hepcidin response during 20-day high-altitude exposure in energy-deficient men [82]. The subjects achieved increased hematocrit and reduced their hepcidin and ferritin levels, indicating that the hypoxic stimuli over time decreased their hepcidin response to make iron more available when the energy supply was low. A single training session has been shown to peak hepcidin responses 3 h after the session [11]. Badenhorst et al. found that a 3-hour hypoxic recovery period [42] and training in hypoxia over time reduced the hepcidin response [11,32,38,75]. Conversely, Govus et al. found that a single session in acute hypoxia did not appear to alter hepcidin responses compared to those who trained in normoxic environments [83]. Goto et al. found similar results in a study of men running in hypoxic and normoxic environments, finding no significant difference in hepcidin responses between the groups during a single endurance session [84]. This shows inconclusive effects of hypoxia on the hepcidin response from training over time, during recovery, in terms of nutritional status, and in single training sessions. Further studies are needed to determine the effects of hypoxia on hepcidin.

Sufficient ferritin levels were reached in studies by both Okazaki et al. and Garvican-Lewis et al. [32,38], ensuring an increase in VO_2max_ and hemoglobin after training in hypoxia, hence the increased endurance capacity. Hepcidin alteration may also be a contributing factor to this increase during the hypoxic stimulus, possibly due to HIF and/or increased erythropoietic mechanisms that can downregulate hepcidin levels [77,78,79]. Studying interventions that can increase the utilization of existing iron in the body would be interesting, such as in cases with FID or when hepcidin levels are high in iron-sufficient athletes. We would rather try to increase the utilization of existing iron to the erythropoiesis by altering hepcidin levels and its response to training. For instance, Hennigar et al. observed a reduced hepcidin response under hypoxic stimuli in energy-deficient men [82], suggesting a potential way to manipulate the diet without affecting performance, only reducing the hepcidin response. Skarpanska-Stejnborn et al. gave athletes cranberry extract supplementation for six weeks and found an attenuated hepcidin response three hours after a 2000 m rowing test [85]. Cranberries have been shown to have anti-oxidant effects [86] and can possibly contribute to reducing the inflammation that occurs due to physical exercise. Domìnguez et al. suggested, in their review of post-exercise expression of serum hepcidin, that cranberry flavonoid supplementation could attenuate the hepcidin response after training [11]. This gives us another factor that requires more study to determine if there is a possible effect on athletes’ iron status and the use of preexisting iron. As a result, multiple factors have been demonstrated to regulate hepcidin, which can vary depending on an athlete’s training regime, and which can affect their iron status. However, iron levels are likely to be greater determining factor of performance than hepcidin. Most studies using a ferritin cut-off of around 30 ng/mL have found significant differences in performance between iron deficiency and sufficiency. Increased iron levels in iron-deficient athletes or athletes at altitude have led to improved performance within ferritin levels ranging from 30 to 99 ng/mL [32,33,39,66].

The correlation between hemoglobin status and physical performance is significant, but the included studies did not delve further into how performance is affected, apart from the general consequence of reduced ability to transport oxygen to the working muscles when hemoglobin levels fall below 130/120 g/L in men/women. Mostly, they looked at which factors increased or decreased hemoglobin levels. The studies involving iron supplementation resulted either in increased hemoglobin levels [38] or ensured that they were not reduced because of physical strain [35,41]. Two studies observed an increase in physical abilities correlated with an increase in hemoglobin [32,39]. Okazaki et al. found that increased hemoglobin levels corresponded to an increase in VO_2max_, and highlighted this process as a part of the increased endurance capacity after altitude training, provided there were sufficient iron stores [32]. Those who trained at sea-level, or had deficient iron stores, did not experience increased hemoglobin or VO_2max_. Della Valle et al. correlated an increased VO_2peak_ to the rowers’ increases in hemoglobin, after iron supplementation, in IDNA females [39]. Only one of the studies involving iron supplementation demonstrated no increase in hemoglobin values [40]. Woods et al. administered IV iron to iron-sufficient athletes (serum iron 30–99 ng/mL), which resulted in an increase in ferritin levels but no increase in hemoglobin levels in both the control and iron-supplemented groups [40]. They also did not observe an improvement in VO_2max_, except in the few athletes who had an initial lower iron status. This might indicate that iron supplementation itself is not enough to increase hemoglobin levels in iron-sufficient athletes under normal circumstances. This will depend on different factors, like training stimulus and iron and hepcidin levels, and may be one of the primary reasons why athletes opt to live and train in hypoxic environments.

### 4.4. Other Markers for Optimization

Kasproviz et al. and Mielgo-Ayuso et al. demonstrated that vitamin D supplementation could prevent a decline in both iron and hemoglobin levels [36,44]. They used the threshold of 25(OH)D < 30 ng/mL for deficiency, finding significant results with regard to physical performance, highlighting impaired oxygen transport as a consequence of iron deficiency. Ensuring that vitamin D levels remain above this threshold would possibly help athletes prevent a decline in iron and hemoglobin levels because of training, especially considering the possible interaction between vitamin D and EPO, and the co-occurrence of vitamin D deficiency and anemia. Therefore, 25(OH)D is an important blood marker to consider for optimizing iron status in athletes. Shuler et al. conducted a review of the benefits of vitamin D on athletes generally, examining factors beyond its correlation with iron status and anemia [87]. They highlighted that the maximum benefits of vitamin D supplementation were achieved in athletes who exceeded the 30 ng/mL limit of 25(OH)D, with increasing benefits up to 50 ng/mL. Thus, they concluded that 50 ng/mL is required for optimal athletic benefits. Athletes may consider aiming to maintain vitamin D levels above 30 ng/mL and target 50 ng/mL, for optimal iron status and to avoid fractures, inflammation, and musculoskeletal pain [88,89,90].

Deficiency of vitamin B12 can reduce endurance and cause anemia [91,92]. Considering the significant correlation between vitamin B12 and hemoglobin levels in the study by Krzywánsk et al., athletes should also pay attention to vitamin B12 levels. A potential target range could be 400–700 pg/mL, as suggested by the study, to support hemoglobin synthesis and improve red cell markers [43]. However, they emphasized that there are limited studies regarding red blood cell parameters and vitamin B12 in athletes, indicating the need for further research to determine optimal vitamin B12 levels for athletes.

Minor positive benefits of iron supplementation on athletes’ recovery were found in this review. Còrdova et al. found lower cortisol levels when supplementing iron-sufficient male cyclists with iron during a three-week stage race [35]. Cortisol, as an important catabolic hormone for energy mobilization, is usually increased with physical strain and muscle damage [93,94]. High cortisol levels indicate a higher level of stress and fatigue on the body, and hence the need for recovery. Còrdova et al. reported lower cortisol responses in athletes given iron supplementation, which indicated a lower stress response from the long and hard demand on the body. Under high physical loads, such as longer races and repeated high efforts with limited amount of rest, recovery between efforts plays a critical role in maintaining performance. In this context, iron supplementation contributed to improved recovery, and could be something for athletes to consider when undergoing high physical strain, with short recovery periods between efforts. Further studies are warranted to explore iron’s effects on recovery and cortisol levels comprehensively.

## 5. Conclusions

Iron deficiency frequently occurs in athletes, especially in endurance athletes. The effects of IDNA, and suboptimal iron status on physical performance remain largely unknown. The protocol for optimizing athletes’ iron status is not set and could possibly be a contributing factor for optimization of physical performance. This review has revealed that iron supplementation has the most pronounced impact on physical performance in athletes with lower ferritin levels, demonstrating significant improvements in iron-deficient athletes while offering limited benefits to athletes with sufficient iron stores. 

Clear guidelines for narrower and more optimal ferritin levels in athletes have not been established, apart from maintaining levels above the threshold for iron deficiency.

Regardless of baseline ferritin levels, iron supplementation seems necessary for optimal erythropoietic response during altitude training. Iron supplementation for the improvement of physical performance should be undertaken with caution because of the possible increased hepcidin response as a result of increased iron levels. How existing iron stores are used for erythropoiesis can vary, as hepcidin is a contributing factor that can affect the use of the existing iron the body. Modifying hepcidin levels may offer a potential avenue to enhance iron utilization in conjunction with training, thereby improving physical performance. While reduced hepcidin responses from training or recovery in hypoxia has been shown, the exact effects of hypoxia on the hepcidin response remain unclear. It is also noteworthy that recent research suggests that maintaining adequate energy and carbohydrates in the diet seems to be an important factor in avoiding increased hepcidin levels after training, which athletes and coaches should pay attention to. Other factors, such as sufficient vitamin D and vitamin B12 levels, can possibly contribute to improved iron status and prevent anemia.

## Figures and Tables

**Table 1:** Summary of the articles in this review with methods, results, and which impact the results can have on performance.

Author	Population	Study	Experimental Protocol	Main Outcomes	Potential Conclusions
**Okazaki et al.** **[32]**	19 + 39 runners	Retro- and prospective study	4 week training camp at 2500 m altitude in athletes with low (*n* = 9) and normal (*n* = 10) ferritin. Iron-sufficient athletes in identical altitude exposure (*n* = 26), against control group (*n* = 13) at sea level. VO_2max_ and RVC measured before/after. 44–306 mg iron given with Vitamin C	Only athletes with normal ferritin levels increased RCV and VO_2max_ after 4 weeks altitude. Supplementation normalized low ferritin levels, maintained normal levels during altitude. RVC and VO_2max_ only increased in altitude group. Daily iron requirement 1.9 mg/day for men and 2.3 mg/day for women at sea level +4.9 mg/day at altitude.	VO_2max_ increase in iron-sufficient athletes exposed to altitude. No optimal response with low ferritin levels. Normalized iron stores prior to altitude training are important for erythropoietic adaptation to hypoxic stimulation. Recommends Se-Fe 40–90 ng/mL before 4-week altitude.
**Córdova et al.** **[35]**	18 male cyclists	RCT	Effect of iron supplementation during a 3-week stage race on hematological status, S-cortisol, and muscle damage. IG (*n* = 9) given 2 × 40 mg iron/day, CG (*n* = 9) cyclists as controls. Blood test 1 week before and at end of race: S-iron, ferritin, Hb, ht, TIBC, TSAT, sTrF, CK, LDH, cortisol.	80 mg/day of iron prevented decrease in iron, ferritin, Hb, and ht compared to CG. More decreased cortisol levels in supplementation group. No difference in muscle damage, but hematological levels associated with muscle damage biomarkers.	Iron supplementation (80 mg/day) can be considered in athletes with hard physical load to prevent decline in iron, and then reduced performance. Can cause lower levels of effort-stress on the body.
**Kasprowicz et al.** **[36]**	20 ultra-marathon runners	RCT	Vitamin D baseline and its impact on post-exercise S-iron, IL-6 and hepcidin response. VD (*n* = 10) given 10,000 UI vitamin D/day, CG (*n* = 10) given placebo, 2 weeks prior to 100 km run. Blood tests prior to supplementation, before the run, after 100 km, and 12 h after finish.	Higher vitamin D levels in VD group. No difference in hepcidin and IL-6 response, but VD had less reduction in iron immediately after the run. Correlation between vitamin D and erythropoiesis. Co-occurrence of vitamin D deficiency and anemia.	Vitamin D status can affect iron metabolism: high doses can inhibit post-exercise reduction of iron. Suggested 25(OH)D cut > 30 ng/mL. Supplementation doses not justified.
**McCormick et al.** **[37]**	16 runners	Crossover study	Iron absorption after training in the morning vs. the afternoon, in runners with Se-Fe < 50 ng/mL. Iron isotopes given in standardized meal after a 90 min 65% run in the morning, or afternoon. Blood tests: IL-6, hepcidin, Se-Fe, Hb and erythrocyte iron-incorporation before, immediately after, 3 h after, and after 14 days.	Increased IL-6 after training, hepcidin increased 3 h after training, and showed diurnal tendency; larger increase when training in the afternoon. Iron best absorbed after exercise in the morning compared to the afternoon, and at rest.	Endurance athletes can potentially increase iron absorption from the diet by consuming iron shortly after exercise in the morning.
**Garvican-Lewis et al.** **[38]**	34 endurance athletes (runners, cyclists, triathletes)	RCT	Hb and erythropoietic response with iron supplementation, iv or orally, during LHTL (3000 m) simulation. Non-anemic endurance athletes were given intavenous or oral iron, or placebo, 2 weeks before, and for 3 weeks LHTL. Blood tests: Hb, ferritin, iron, sTFr, TSAT. Hepcidin, erythroferrone.	Hb increased 3.2% in oral, 3.7% in IV supplementation, and no increase in placebo, after 21 days. Ferritin increased more in intravenous than oral. Stable ferritin levels indicate supplemented iron is used for erythropoiesis. Iron supplementation only increased Hb during hypoxic stimulus. VO_2peak_ increased with IV supplementation.	Iron supplementation is necessary for optimal erythropoietic adaptation to hypoxic exposure. IV iron gave no additional benefit over oral iron in non-anemic athletes.
**DellaValle et al.** **[39]**	40 female rowers	RCT	Effects of iron supplementation in non-anemic women. 2 × 50 mg iron sulfate (IG *n* = 21) or placebo (CG *n* = 19) given with citrus juice for 6 weeks. Defined IDNA (Se-Fe < 20 ng/mL) or normal (Se-Fe > 20 ng/mL) prior to intervention. Blood tests: Hb, Ht, Se-Fe and sTfR at baseline/endpoint. VO_2peak_, EF and blood lactate after 6 weeks.	IG improved Se-Fe, had slower lactate response, and showed better energy expenditure and EF compared to placebo. Both groups improved VO_2peak_. Rowers with lower Se-Fe had better improvement in Fe-stores. IDNA affects lactate response without anemia.	Improved iron stores, lactate, and EF during endurance training with iron supplementation. Those with lower iron stores at baseline benefit more from supplementation. Supplementation may increase benefits of endurance training when in risk for iron deficiency.
**Woods et al.** **[40]**	14 distancerunners	RCT	Effect of IV iron in runners with Se-Fe 30–100 ng/mL for 6 weeks training. IG (*n* = 7) received IV iron, CG (*n* = 7) received placebo. 3 injections over 4 weeks. Tested: 3000 m TT and 10 × 400 m monitored session at start and following each injection. Hb in week 0 and 6. TMD (mood) and TFS (fatigue) every second week until week 6.	IG increased ferritin to double, no increase in Hb. Increased TFS and TMD in IG compared to placebo. No improvement in physical performance in both groups.	4 weeks intravenous iron did not increase physical performance in athletes with no clinical iron deficiency, training under normal circumstances for 6 weeks. Improved mood and fatigue, can increase quality in training, which can increase performance.
**Mielgo-Ayuso et al.** **[41]**	22 female volleyball players	Follow up study	Investigate if the benefits of an 11 week oral iron supplementation protocol lasted after cessation, in the remaining 8 weeks of the season. IG (*n* = 11) 325 mg/day iron, CG (*n* = 11). Adequate iron >100 ng/mL, functional iron deficiency 30–99 ng/mL, absolute deficiency < 30 ng/mL, anemia Hb < 12 g/dL. Blood tests: Hb, Se-Fe, TSAT, Ht, S-iron at W0, 11, 21 and 29. Strength tests: press, squat, clean, pullover and total mean strength.	IG maintained iron status after 11 weeks, CG decreased. IG back to baseline 10 weeks after cessation (W21) and did not maintain for the rest of the season (W29). IG increased strength more than CG in the first 11 weeks. Opposite in the following 8 weeks (W21-29): CG had better ferritin and Hb than IG, and increased strength.	Benefits of iron supplementation will not last for multiple months after cessation in female volleyball players. Suggesting sustained supplementation during the competitive season to keep iron stores sufficient. Blood assessment to decide if supplementation is needed and to avoid toxicity.
**Badenhorst et al.** **[42]**	10 male runners	Crossover study	Assess the influence of a 3 h hypoxic (2900 m) recovery period post-running on exercise inflammation (IL-6), Se-Fe and iron, hepcidin, and EPO. 8 × 3 min run at 85% Blood tests: pre/post run, after 3 h and 24 h recovery in hypoxia.	Similar increase in IL-6 immediately after training. Hepcidin levels elevated 3 h post running, but lower in hypoxic recovery group. No difference in EPO, S-iron, or ferritin between different recovery environments.	Hypoxic recovery, after intense endurance exercise, can lower the hepcidin response to training. Can be useful to potentially increase acute iron absorption from the diet.
**Krzywànsk et al.** **[43]**	243 track and field athletes	Prospective study	Asses vitamin B12 status and its influence on red blood cell parameters in elite athletes. SG = 189 and EG = 54 athletes, 1131 blood samples over 6 years. 34% used B12-injections. Red blood cell parameters: Hb, Ht, MCV, MCH and vitamin B12. Deficiency cut vitamin B12 < 197 pg/mL.	Average vitamin B12 739 pg/mL in SG, 881 pg/mL in EG, no deficiencies. Significant relationship between B12 and Hb: increase in Hb from low B12 levels up to 400 pg/mL, and no change in Hb from 700 pg/mL and onwards.	Vitamin B12 range between 400–700 pg/mL might favor Hb synthesis in athletes. Should be monitored regularly and consider oral supplementation < 400 pg/mL.
**Mielgo-Ayuso et al.** **[44]**	36 male rowers	RCT	8 weeks vitamin D supplementation and its effect on hematological status, testosterone, and cortisol values. Vitamin D group (*n* = 18) 3000 IU/day, control group (CG *n* = 18) given placebo. Blood tests at start/end: Hb, Ht, iron, ferritin, transferrin, 25(OH)D, testosterone, cortisol. Deficiency cut 25(OH)D < 30 ng/mL	8-week vitamin D supplementation prevented a reduction in Hb, transferrin and Ht, improved 25(OH)D levels. No improvement in muscle recovery based on testosterone and cortisol levels, but 25(OH)D can be a predictor of anabolic and catabolic hormones.	Vitamin D supplementation can prevent a reduction in hematological parameters, and potentially contribute to a better transport of oxygen.

Abbreviations: CG = control group, CK = creatin kinase, EG = endurance group, EPO = erythropoietin, erythroferrone = regulates iron by inhibiting hepcidin, ferritin = primary iron storage protein, hepcidin = main regulator of iron homeostasis(inhibitor), Ht = hematocrit: proportion of red blood cells in blood, Hb = hemoglobin: protein carrying oxygen in red blood cells, IG = intervention group, IL-6 = interleukin 6, IV = intravenous, LDH = lactate dehydrogenase(enzyme), MCH = mean corpuscular hemoglobin: average hemoglobin in red blood cells, MCV = mean corpuscular volume: average volume of red blood cells, RCV = red blood cell volume, SG = strength group, sTfR = transferrin receptor: membrane receptor involved in iron supply to the cell by the binding of transferrin, TIBC = total iron binding capacity: bloods capacity to bind iron with transferrin, transferrin = main carrier protein of iron, TSAT = transferrin saturation: ratio of serum iron to TIBC, VD = vitamin D group, vitamin = vitamin, VO_2max_ = maximum oxygen consumption that can be achieved during physical exertion, VO_2peak_ = the highest VO_2_ value attained on a test were intensity increases until the individual reaches volitional exhaustion, 25(OH)D = measure on vitamin D stores.3.2. Population characteristics.

## Data Availability

Data is available on request.

## Supplementary Materials

The following supporting information can be downloaded at: https://www.mdpi.com/article/10.3390/life13102007/s1, Algorithm of the systemic search performed in Pubmed.
