# Correction: Solberg, A.; Reikvam, H. Iron Status and Physical Performance in Athletes. *Life* 2023, *13*, 2007

**DOI:** 10.3390/life16010056

**Published:** 2025-12-30

**Authors:** Andrea Solberg, Håkon Reikvam

**Affiliations:** 1 Faculty of Medicine, University of Bergen, 5007 Bergen, Norway; andrea.solberg@student.uib.no; 2 Institute of Clinical Science, Faculty of Medicine, University of Bergen, 5007 Bergen, Norway; 3 Clinic for Medicine, Haukeland University Hospital, 5009 Bergen, Norway

In the original publication [1], there was an error in the Discussion section.

“When comparing the importance of iron supplementation in iron-deficient versus iron-sufficient athletes in this review, clear physical improvements were observed in supplemented athletes with baseline ferritin levels below 30 mg/mL. This confirms the importance of monitoring and providing supplementation to athletes who are susceptible to iron deficiency. With regard to finding out the effects iron supplementation can have on athletes with ferritin levels above 30 mg/mL, no major results stand out here, although positive correlations were found.”

And the ferritin level mentioned in the text should be 30 ng/mL, not 30 mg/mL. 

A correction has been made to 4. Discussion, 4.2. Iron Deficiency vs. Iron Sufficiency, Paragraph 2:

“When comparing the importance of iron supplementation in iron-deficient versus iron-sufficient athletes in this review, clear physical improvements were observed in supplemented athletes with baseline ferritin levels below 30 ng/mL. This confirms the importance of monitoring and providing supplementation to athletes who are susceptible to iron deficiency. With regard to finding out the effects iron supplementation can have on athletes with ferritin levels above 30 ng/mL, no major results stand out here, although positive correlations were found.”

The authors state that the scientific conclusions are unaffected. This correction was approved by the Academic Editor. The original publication has also been updated.
