# Supplementary material for: Iron Status and Physical Performance in Athletes
Source: Life (Basel). 2023 Oct 2;13(10):2007. doi: 10.3390/life13102007 (PMC10608302; doi:10.3390/life13102007)
Supplement: Supplementary file 1 [file life-13-02007-s001.zip › life-2598920-supplementary.pdf]

The search was performed in Pubmed.

Dato: 15.06.2023

#1
Search: **"Hemoglobins"[Mesh:NoExp] OR "Iron"[Mesh:NoExp] OR "Iron Deficiencies"[Mesh] OR "Hepcidins"[Mesh] OR "Ferritins"[Mesh:NoExp]**
190,894

#2
Search: **Iron[Title/Abstract] OR ferritin*[Title/Abstract] OR transferrin*[Title/Abstract] OR hepcidin*[Title/Abstract] OR haemoglobin[Title/Abstract] OR hemoglobin[Title/Abstract]**
447,408

#3 (#1 OR #2)
Search: **("Hemoglobins"[Mesh:NoExp] OR "Iron"[Mesh:NoExp] OR "Iron Deficiencies"[Mesh] OR "Hepcidins"[Mesh] OR "Ferritins"[Mesh:NoExp]) OR (Iron[Title/Abstract] OR ferritin*[Title/Abstract] OR transferrin*[Title/Abstract] OR hepcidin*[Title/Abstract] OR haemoglobin[Title/Abstract] OR hemoglobin[Title/Abstract])**
494,760

#4
Search: **"Athletes"[Mesh:NoExp] OR "Athletic Performance"[Mesh]**
77,747

#5
Search: **Athlete*[Title/Abstract] OR athletic[Title/Abstract]**
77,450

#6 (#3 OR #4)
Search: **("Athletes"[Mesh:NoExp] OR "Athletic Performance"[Mesh]) OR (Athlete*[Title/Abstract] OR athletic[Title/Abstract])**
130,159

#7 (#3 AND #6)
Search: **(("Hemoglobins"[Mesh:NoExp] OR "Iron"[Mesh:NoExp] OR "Iron Deficiencies"[Mesh] OR "Hepcidins"[Mesh] OR "Ferritins"[Mesh:NoExp]) OR (Iron[Title/Abstract] OR ferritin*[Title/Abstract] OR transferrin*[Title/Abstract] OR hepcidin*[Title/Abstract] OR haemoglobin[Title/Abstract] OR**

hemoglobin[Title/Abstract])) AND (("Athletes"[Mesh:NoExp] OR "Athletic Performance"[Mesh]) OR (Athlete*[Title/Abstract] OR athletic[Title/Abstract]))
2,676

#8 (#3 AND #6, Filters: in the last 10 years)
Search: **(("Hemoglobins"[Mesh:NoExp] OR "Iron"[Mesh:NoExp] OR "Iron Deficiencies"[Mesh] OR "Hepcidins"[Mesh] OR "Ferritins"[Mesh:NoExp]) OR (Iron[Title/Abstract] OR ferritin*[Title/Abstract] OR transferrin*[Title/Abstract] OR hepcidin*[Title/Abstract] OR haemoglobin[Title/Abstract] OR hemoglobin[Title/Abstract])) AND (("Athletes"[Mesh:NoExp] OR "Athletic Performance"[Mesh]) OR (Athlete*[Title/Abstract] OR athletic[Title/Abstract]))** Filters: **in the last 10 years**
1,150

#9 (#3 AND #6, Filters: in the last 10 years, Adult: 19-44 years)
Search: **(("Hemoglobins"[Mesh:NoExp] OR "Iron"[Mesh:NoExp] OR "Iron Deficiencies"[Mesh] OR "Hepcidins"[Mesh] OR "Ferritins"[Mesh:NoExp]) OR (Iron[Title/Abstract] OR ferritin*[Title/Abstract] OR transferrin*[Title/Abstract] OR hepcidin*[Title/Abstract] OR haemoglobin[Title/Abstract] OR hemoglobin[Title/Abstract])) AND (("Athletes"[Mesh:NoExp] OR "Athletic Performance"[Mesh]) OR (Athlete*[Title/Abstract] OR athletic[Title/Abstract]))** Filters: **in the last 10 years, Adult: 19-44 years**
